# NICE at 25: A Quarter Century of Evidence, Values and Innovation in Health. By P. Littlejohns and K. Syrett (eds.), Abingdon: Routledge, 2025. 203 pp. £130 (hardback); £33.99 (ebook). ISBN: 978‐1‐003‐50126‐8

**DOI:** 10.1111/1467-9566.70065

**Published:** 2025-07-03

**Authors:** Gowree Balendran

**Affiliations:** ^1^ Faculty of Medicine Keele University Keele Staffordshire UK

Marking the 25th anniversary of the National Institute for Health and Care Excellence (NICE), this text aims to provide readers with a ‘fuller understanding’ of NICE's evolution. Beginning with a history of NICE, key issues informing NICE's work are analysed from eight social science perspectives in separate chapters: health economics, political science, health policy, ethics, philosophy, public health, patient and public involvement and law. The main contribution of the book is its account of how NICE has thrived as an independent evidence‐based decision‐maker and evolved to become so successful. NICE at 25 is interesting for its carefully selected historical content, critical analyses of key principles and insights into NICE's evolution; however, there are some significant omissions, although several authors have been closely connected with NICE from its inception in 1999. Despite its relevance, a strong sociological perspective is missing from these analyses, which could have offered a deeper understanding of NICE's role as a cost‐effectiveness regulator. My review focuses on two key issues: NICE's political ‘independence’ and its emerging role in promoting pharmaceutical innovation.

Chapter 1 outlines NICE's history but omits key events, such as the formation of the PICTF in 1999, which gave pharmaceutical executives privileged access to ministers following NICE's rejection of Relenza. It also fails to note NICE's reversal of that decision within a year, despite expert objections, or the Government's broader commitment to creating a pro‐pharmaceutical regulatory environment (PICTF 2001). These omissions limit readers' understanding of NICE's evolving role and its entanglement with industry interests.

Chapter 2 is an excellent evaluation of NICE's evolution within its political environment by Claxton et al., which reveals how NICE's decisions on new medicines have reduced overall population health and increased health inequalities. These authors acknowledge that ‘complexities’ of the political environment in which NICE operates are ‘limited in their transparency’ but do not attempt to describe that environment. Understanding the political context is essential to understand NICE's role as an ‘independent’ evidence‐based decision‐maker; hence, transparency is crucial.

Chapter 3 presents NICE as politically independent but overlooks the pharmaceutical industry's significant influence on government policy through bodies such as PICTF and the NICE‐ABPI Industry Council. It also fails to consider NICE's role in advancing pro‐industry government objectives, as formalised in the NICE/ABPI Partnership Agreement. Although highlighting NICE's resilience under small‐government ideology, the authors neglect how funding cuts led to increased reliance on industry fees for appraisals—rising from 5.2% of NICE's income in 2019–20 to 12.9% in 2022–23—undermining its independence by embedding market principles into its regulatory function (Abraham* and Balendran* 2025).

In Chapter 4, Weale argues that NICE's core cost‐effectiveness paradigm remains unchanged. The shift from ‘social values’ to ‘principles’ reflects a change in public presentation to align with political priorities, not a transformation of NICE's role. The classical use of incremental cost‐effectiveness ratios (ICERs), quality‐adjusted life years (QALYs) and opportunity costs, framed by social values, continues to function as a ‘fourth hurdle’ for pharmaceutical manufacturers. Although Charlton (Chapter 5) and Wilson (Chapter 6) highlight NICE's greater emphasis on innovation, NICE's role as facilitator of innovation is not new. Analysis of NICE's technology appraisal decisions from its inception revealed that NICE approved the vast majority of new pharmaceutical products (Abraham* and Balendran* 2025). In its first year, NICE approved all pharmaceutical appraisals; in its first seven years (1999–2006), only 5% of NICE's pharmaceutical appraisals resulted in rejection. NICE has balanced its gatekeeper role with its role as facilitator of pharmaceutical innovation from its inception. What has changed as a result of neo‐liberalism is the visibility of NICE's role as facilitator of innovation.

The book concludes with the clear message that NICE thrived by heeding stakeholders' concerns and evolving methods accordingly; hence, NICE must heed concerns that it could lose its status, identity and legitimacy unless a cultural change occurs in politics/policymaking to ensure complex healthcare decisions are based on ‘solid grounding in evidence’. In my opinion too, it is crucial to its credibility that NICE prioritises public health interests, especially in contexts where these conflict with the interests of commercial industry. NICE at 25 is recommended for medical sociology researchers, healthcare professionals and policy professionals and is essential reading for anyone researching pharmaceutical cost‐effectiveness regulation.

## Author Contributions

The author is solely responsible for all aspects of the manuscript.

## Data Availability

Data sharing is not applicable to this article as no new data were created or analyzed in this study.
